# Rapid Assembly of Block Copolymer Thin Films via Accelerating the Swelling Process During Solvent Annealing

**DOI:** 10.3390/polym17091242

**Published:** 2025-05-02

**Authors:** Tian-en Shui, Tongxin Chang, Zhe Wang, Haiying Huang

**Affiliations:** 1 College of Materials Science and Engineering, Changchun University of Technology, Changchun 130012, China; 2 State Key Laboratory of Polymer Physics and Chemistry, Changchun Institute of Applied Chemistry, Chinese Academy of Sciences, Changchun 130022, China; 3 Key Laboratory of Advanced Functional Polymer Membrane Materials of Jilin Province, Changchun 1300122, China; 4 College of Chemistry and Life Science, Changchun University of Technology, Changchun 130012, China

**Keywords:** block copolymer, thin film, self-assembly, solvent annealing, long-range order

## Abstract

Block copolymer (BCP) lithography is widely regarded as a promising next-generation nanolithography technique. However, achieving rapid assembly with defect-free morphology remains a significant challenge for its practical application. In this study, we presented a facile and efficient solvent annealing method for fabricating well-ordered BCP thin films within minutes on both flat and topographically patterned substrates. By accelerating the swelling process, rapid film swelling was observed within just 10 s of annealing, leading to well-ordered morphologies in 1~3 min. Furthermore, we systematically investigated the influence of swelling ratio (SR) on film morphology by precisely tuning solvent vapor pressure. For cylinder-forming poly(styrene-block-2-vinylpyridine) (PS-b-P2VP) films, we identified three distinct SR-dependent ordering regimes: (I) Excessive SR led to a disordered morphology; (II) near-optimal SR balanced long-range and short-range orders, and a slight increase in SR enhanced the long-range order but introduced short-range defects. (III) Insufficient SR failed to provide adequate chain mobility, limiting long-range order development. These findings highlight the critical role of SR in controlling defect density in nanopatterned surfaces. Long-range-ordered BCP nanopatterns can only be achieved under optimal SR conditions that ensure sufficient chain mobility. We believe this rapid annealing strategy, which is also applicable to other solvent-based annealing systems for BCP films, may contribute to next-generation nanolithography for microfabrication.

## 1. Introduction

Block copolymer (BCP) thin film can spontaneously self-assemble into highly tunable ordered nanostructures, making them a promising nanopatterning technology and a strong candidate for next-generation lithography, surpassing traditional photolithography [1,2,3,4,5,6,7,8,9,10,11,12,13,14,15]. Typically, thermal or solvent annealing is employed to facilitate BCP self-assembly and achieve well-ordered nanostructures [16,17,18]. However, thermal annealing is limited by the low degradation temperature (*T*_d_) of organic polymers, which imposes an upper bound on the annealing temperature (*T*_a_), inevitably resulting in prolonged annealing times. To align with conventional lithography production processes, reducing annealing time is crucial, particularly for BCPs with small characteristic periods and a high Flory–Huggins interaction parameter (*χ*) [19,20]. For high-*χ* BCP, assembly kinetics is further hindered by slow chain diffusivity across phase interfaces [21,22]. Although rapid BCP thin film assembly has been demonstrated by annealing at temperatures significantly exceeding *T*_d_, its long-term reliability remains uncertain and requires further validation [23,24,25,26]. Solvent annealing offers an alternative approach to enhancing BCP assembly kinetics, as solvent molecules act as both plasticizers and diluents, significantly reducing the glass transition temperature (*T*_g_) and polymer viscosity at room temperature (Supporting Information S1) [27]. However, conventional solvent annealing still requires several hours to achieve high-quality BCP morphologies, presenting a significant limitation for practical applications.

Recently, several advanced solvent annealing strategies have been developed to realize the fast assembly of BCP thin films [28,29,30,31,32,33,34,35,36,37,38,39,40,41,42,43,44,45,46], such as microwave annealing [28,29,30,31], solvothermal annealing [32,33,34,35,36], direct immersion annealing [37,38,39], swollen PDMS-assisted annealing [40], and warm spin-coating with high-boiling-point solvent [41,42,43], among others [44,45,46]. However, these methods still face key challenges, such as limited reproducibility, precise control over solvent vapor pressure, achieving high swelling speed, and preventing solvent condensation. A more universal, cost-effective, and controllable annealing strategy is still highly desirable for the rapid fabrication of long-range-ordered BCP nanopatterns. The solvent annealing process can be conceptually divided into three stages: (1) swelling of the BCP thin film, (2) self-assembly of BCP in the swollen state, and (3) removal of the swelling solvent. BCP reorganization occurs only when the glass transition temperatures (*T*_g_) of the polymers are reduced below the annealing temperature, facilitated by solvent-induced plasticization. Since the optimal swelling ratio (SR) for a given annealing system is fixed, accelerating the initial swelling rate, which reduces the time required for BCP chain movement, is crucial for minimizing the overall annealing time under fixed conditions.

Herein, we achieved rapid swelling and fast assembly of BCP thin films within seconds by confining the annealing process within a flat container with a high surface-to-volume ratio. This setup allowed the precise tuning of vapor pressure, enabling controlled swelling ratio (SR, swollen film thickness *H* divided by initial film thickness *H*_0_, i.e., *H*/*H*_0_) [35]. Additionally, this method required only a minimal amount of annealing solvent, making it an environmentally friendly approach. Since the swollen film thickness is highly sensitive to solvent vapor pressure, we systematically investigated the influence of the SR on BCP morphology ordering across various BCP systems and template types. Furthermore, we demonstrated that this annealing strategy was broadly applicable, enabling precise control over the long-range ordering of BCP nanostructures under optimal SR conditions.

## 2. Materials and Methods

### 2.1. Materials

Poly(styrene-*block*-2-vinylpyridine) (PS_62k_-*b*-P2VP_26k_, PDI: 1.13, PS_30k_-*b*-P2VP_12.5k_, PDI: 1.06), poly(styrene-*block*-tert-butyl acrylate) (PS_67k_-*b*-PtBuA_32k_, PDI: 1.04), and poly(butyl acrylate) (PtBuA 23 kDa, PDI: 1.15) were purchased from Polymer Source, Inc., Canada. Toluene, tetrahydrofuran (THF), and chloroform (analytical pure) were purchased from Sinopharm Chemical Reagent Co., Ltd., Shanghai, China. Sylgard 184 was purchased from Dow Corning Co., Ltd., Singapore. Toluene was dried with anhydrous magnesium sulfate, and THF was dried with calcium hydride before use. Other materials were used as received.

### 2.2. Solvent Annealing of BCP Thin Films

Firstly, all substrates were treated with piranha solution (H_2_SO_4_/H_2_O_2_ = 7:3) at 120 °C for 30 min, and then they were cleaned thoroughly using sonication in acetone, ethanol, and deionized water, respectively, and dried with nitrogen flow. BCP thin films were then deposited by spin coating from the 0.3~0.5 wt% chloroform solution. The as-spun thin film was placed in the flat PTFE container with a size of 2 cm in diameter and 5 mm in height, keeping 2~3 mm away from the chamber bottom. The annealing container was placed in a homemade drying box at 22 °C with low humidity (~30% RH). A small quantity of annealing solvent (~30 μL) was introduced into the chamber bottom, after which the container was sealed with a square quartz cover. The container was then sealed with a square quartz cover, with spacers made of plastic adhesive tape stuck to two sides of the cover, leaving gaps on the other two sides. This enabled the vapor leakage rate to be tuned with great precision. The thickness of the plastic tape monolayer was measured to be approximately 0.05 mm using a micrometer. The height of the spacer was varied by applying different layers of tape to the quartz cover. The film swelling process was monitored in situ by reflectometry F20-UV (wavelength: 200~1100 nm), and the film thickness was fitted with a three-layer model (silicon substrate, 2 nm native silicon dioxide and polystyrene). Following the annealing process for a given time, the quartz cover was opened immediately, thereby preserving the film morphology through exposure to ambient conditions. To ensure the accurate comparison of the annealing sample at different swelling ratios, each annealing condition was repeated multiple times.

### 2.3. Fabrication of the Patterned Substrate

The silicon grating template was purchased from Shanghai NTI Co. Ltd. (Shanghai, China), with a period of 833 nm, mesa width of 416.5 nm, and trench depth of 300 nm. The silicon template was modified with 1H,1H,2H,2H-perfluorodecyltrimethoxysilane before use. Following this, a PDMS replica was prepared by casting sylgard 184 (weight ratio: 10:1) onto the template and baking at 60 °C for 6 h. Then, polystyrene film was spin-cast onto a silicon substrate with a 300 nm SiO_2_ layer from a 20 mg/mL toluene solution. The grating structure was transferred from the PDMS template to the PS film by soft nanoimprinting at 150 °C, 200 kPa, for 2 h. O_2_ (50 W, 10 Pa, 10 s) plasma and CF_4_ plasma (100 W, 50 Pa, 150 s) were applied to transfer the PS pattern onto the silicon substrates. The patterned substrates were further cleaned with toluene to remove the residual PS molecules.

### 2.4. Fabrication of Pt Nanoarrays on the Annealing BCP Thin Films

The annealed BCP thin films were immersed in 10 mM HPtCl_6_ 0.5% HCl aqueous solution for 10 min and then rinsed with deionized water and dried with N_2_ gas. Oxygen plasma (50 W, 8 Pa, 60 s) etching was applied to remove the BCP template and reduce Pt precursors in the Pt nanoparticles (NPs), leaving the Pt nanowires or nanodots on the substrates.

### 2.5. Characterizations

To observe and analyze the self-assembled BCP morphology, a SPA-300 HV tapping-mode AFM (Seiko Instruments Inc., Japan) and a Hitachi S-4800 field emission scanning electron microscope (SEM) (Hitachi Ltd., Japan) operating at 10 kV were used. The P2VP domains appeared dark, and the PS domain appeared bright in the height images. F20-UV reflectometry was applied to monitor the BCP film thickness in situ with a wavelength range of 200~1100 nm.

## 3. Results and Discussions

**Scheme 1:**
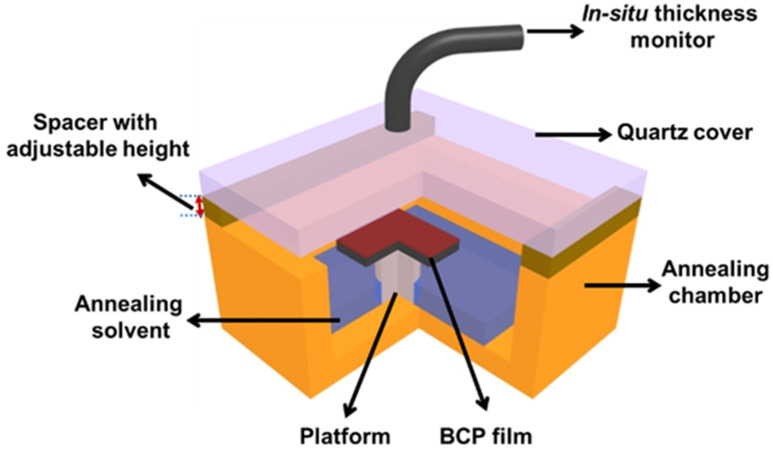
The 1/4 sectional view of the fast-assembly annealing setup: the annealing process was confined within a flat container. A spacer was inserted between the quartz cover and the container to adjust the solvent vapor pressure.

**Figure 1:**
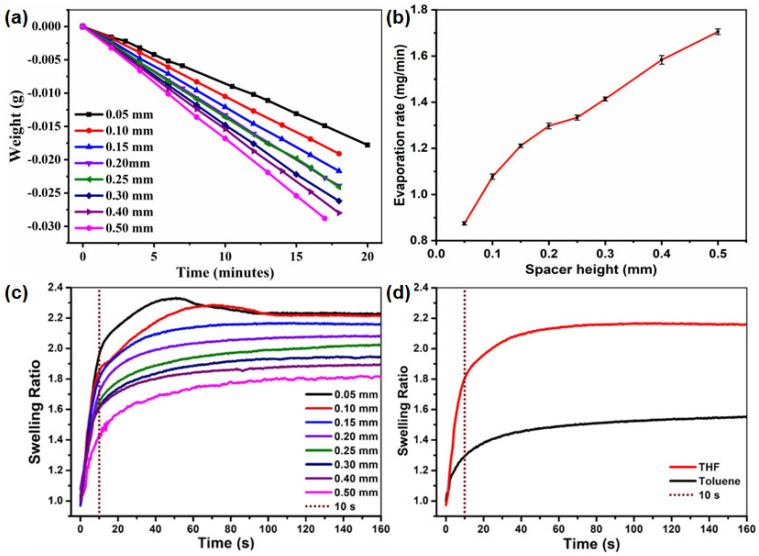
(**a**) The solvent weight change of THF over time. (**b**) The evaporation rate of THF at different spacer heights. (**c**) Swelling profiles of 30 nm PS_62k_-*b*-P2VP_26k_ thin films annealed with THF vapor at different spacer heights. (**d**) Comparison of 30 nm PS_62k_-*b*-P2VP_26k_ thin films annealed with THF and toluene, respectively, at a spacer height of 0.15 mm.

In this work, the annealing process was confined within a flat container with a high surface-to-volume ratio to ensure rapid solvent evaporation and uniform vapor distribution (Scheme 1). The spacer height between the container and the quartz cover was adjustable, allowing for precise control over solvent vapor pressure, which directly correlated with the swelling ratio (SR) of the BCP film. In other words, this design optimized solvent vapor annealing conditions, enabling both rapid swelling and fast BCP film assembly within minutes. Before annealing, we first measured the weight loss of THF over time under different annealing conditions. As shown in Figure 1a, THF evaporation followed a linear trend, indicating a constant evaporation rate. Correspondingly, the evaporation rate could be finely controlled by adjusting the spacer height (Figure 1b). It should be noted that the spacer height was a critical, adjustable parameter that governed solvent evaporation kinetics during annealing, thereby allowing effective tuning of the swelling ratio (defined as the ratio of swollen film thickness *H* to initial film thickness *H*_0_, i.e., *H*/*H*_0_) [35].

To investigate the rapid assembly process, we used cylinder-forming poly(styrene-*block*-2-vinylpyridine) (PS-*b*-P2VP) diblock copolymers with different molecule weights (PS_62K_-*b*-P2VP_26K_ and PS_30K_-*b*-P2VP_12.5K_). As annealing solvents, THF (good solvent for both PS and P2VP) and toluene (preferential affinity for PS than P2VP) were selected to induce parallel and perpendicular cylinder orientations, respectively [7]. Figure 1c–d presents the in situ recorded swelling process of 30 nm PS_62_K-b-P2VP_26_K thin films during annealing with THF or toluene. The films rapidly reached equilibrium swelling within 10 s of annealing (Figure 1c). The swelling ratio of THF exceeded that of toluene due to its higher affinity for both PS and P2VP blocks, which can induce more pronounced swelling behavior in films (Figure 1d). Additionally, for both diblock copolymers annealed in THF, the equilibrium swelling ratio (SR) decreased as the spacer height increased (0.05 mm~0.5 mm), confirming an effective swelling regime under our annealing conditions (Table 1). Notably, the control of BCP film swelling via spacer height demonstrated high reproducibility, further validating the reliability of this approach.

To gain deeper insight into the swelling process, we analyzed the thermodynamics of film swelling. The change in system free energy (ΔG) (1) during swelling can be divided into two components: the condensation of solvent vapor, ΔG_g-l_ (2), and the mixing of solvent with the block copolymer, ΔGmix (3):

(1) $$\Delta G=\Delta G_{g-l}+\Delta G_{\mathrm{mix}}$$

(2) $$\Delta G_{g-l}=G_{l}-G_{g}=G_{g,\;P^{*}}\;-\;G_{g,\;P}=\mathrm{RTln}\left(P^{*}/P\right)$$

(3) $$\Delta G_{\mathrm{mix}}=(\chi_{S-\mathrm{AB}}\varphi_{p}^{2}+\ln(1\;-\varphi_{p})+(1\;-\;N^{-1})\varphi_{p})\mathrm{RT}$$

*G*_l_ and *G*_g_ refer to the free energy of solvent molecules in liquid and gas states, respectively, and *P* and P* are the actual and saturated vapor pressures of the solvent at temperature T. *χ*_S-AB_ refers to the Flory–Huggins interaction parameter between BCP and the solvent. *φ_p_* refers to the volume content of BCP in the swollen film. *N* is the polymerization degree of BCP. Since the vapor pressure *P* in the annealing chamber is always lower than P*, Δ*G*_g−l_ remains positive, thereby hindering the swelling process. However, when the BCP has strong affinity for the annealing solvent, mixing occurs spontaneously, leading to a negative ΔG_mix_, which drives the swelling process. As a result, higher vapor pressure (*P* close to P*) promotes swelling, and modulating *P* allows for the precise tuning of Δ*G*_g−l_ and, consequently, the SR during annealing. In this work, *P* was finely controlled by adjusting the spacer height, thereby achieving the effective regulation of SR (Figure 1c). As swelling progressed, the process transitioned from mixing to dilution, evidenced by the gradual decrease in the swelling rate over time. Swelling ceased once the energy dissipation (ΔG_mix_) was insufficient to counteract the energy input (ΔG_g−l_), at which point the equilibrium SR was reached.

**Figure 2:**
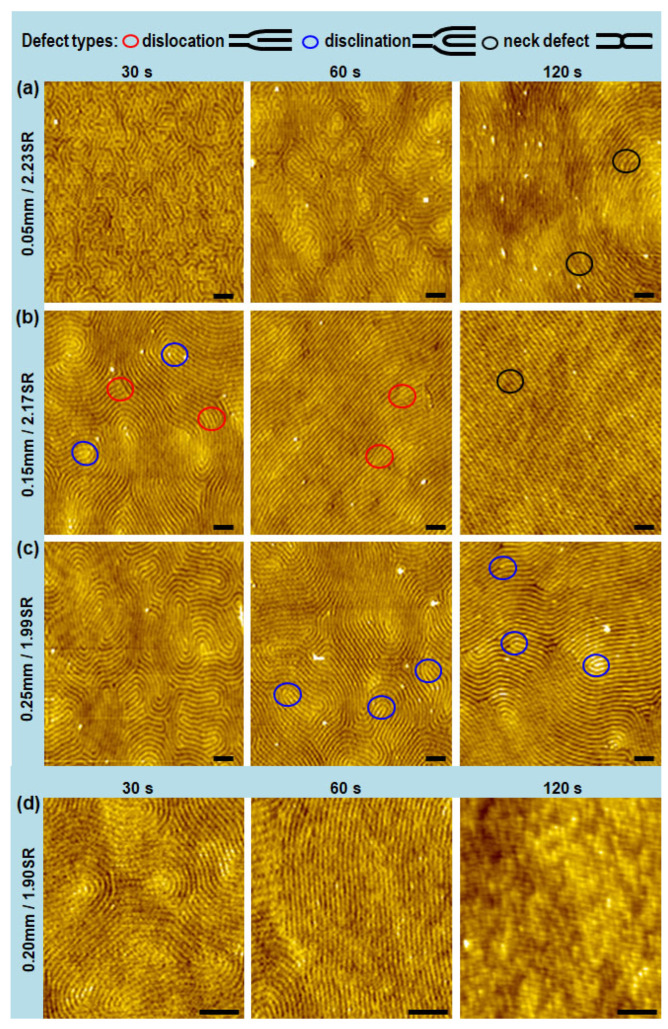
Typical AFM height images of the PS-*b*-P2VP thin films annealed in THF vapor with different SRs for 30 s, 60 s and 120 s, respectively, at 22 °C; (**a**–**c**) 30 nm PS_62k_-*b*-P2VP_26k_ films annealed with different equilibrium SRs; (**d**) 20 nm PS_30k_-*b*-P2VP_12.5k_ films annealed with an equilibrium SR of 1.90. The different colored circles are examples of dislocation (red), disclination (blue), and neck defect (black). Scale bar: 200 nm.

Figure 2 presents the morphology of PS-*b*-P2VP thin films after annealing in THF at different equilibrium SRs for 30 s, 60 s, and 120 s. For PS_62k_-*b*-P2VP_26k_, annealing at an equilibrium SR = 2.23 for 30 s resulted in a disordered structure (Figure 2a), likely due to the rapid attainment of a high SR (Figure 1c), which significantly weakened the phase separation strength (*χN*). After 60 s, P2VP parallel cylinders appeared with numerous disclinations and dislocations. Extending the annealing time to 120 s led to the elimination of most disclinations, although neck defects (bridge-like connections between adjacent cylinders) remained. The formation of neck defects may be driven by the abrupt increase in diffusivity across the cylinders at a high SR. Notably, the Dynamic Self-Consistent Field Theory (DSCFT) simulations did not predict neck defects in the ordered cylinder phase [47], suggesting a small energy difference between defect-free and neck-defect morphologies. Reducing the equilibrium SR to 2.17 (Figure 2b) resulted in PS_62k_-*b*-P2VP_26k_ cylinders with disclinations after 30 s, which were largely eliminated after 60 s, though dislocations remained [48]. After annealing for 120 s, long-range-ordered P2VP parallel cylinders with fewer defects were achieved. Further decreasing the equilibrium SR to 1.99 (Figure 2c) led to short-range-ordered P2VP cylinders with more disclinations, compared to the morphology observed at SR = 2.17. Additionally, the average domain period increased slightly from 46 nm to 50 nm when decreasing the SR from 2.23 to 1.99, which can be attributed to changes in effective segregation strength (*χ*_eff_*N*) [49] (Supporting Information S2). It was demonstrated that the precise control of the SR effectively reduced the annealing time required for BCP self-assembly, and an optimal SR was crucial for achieving long-range order. A parallel experiment was also conducted with PS_30k_-*b*-P2VP_12.5k_. When annealed at an equilibrium SR of 1.90, well-ordered cylinders with minimal defects formed within just 30 s (Figure 2d), while long-range-ordered P2VP parallel cylinders emerged by 60 s and 120 s. The shorter annealing time for the lower molecular weight PS_30k_-*b*-P2VP_12.5k_ can be attributed to its weaker phase separation strength (*χN*) and lower viscosity, both of which enhanced chain mobility at a lower SR. Therefore, with precise control of the SR, the optimum SR for rapid assembly should be 2.17 for PS_62k_-*b*-P2VP_26k_ and 1.90 for PS_30k_-*b*-P2VP_12.5k_.

**Figure 3:**
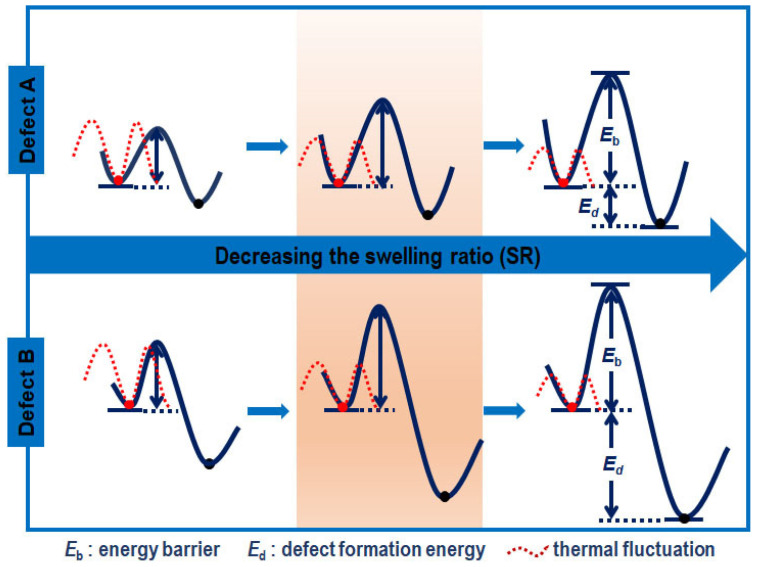
Schematic diagram of the energy landscapes for different defect types. As the SR decreased*,* the thermal fluctuation of the system became weaker, while *E*_d_ and *E*_b_ increased.

The experimental results revealed a significant dependence of both defect type and density on the film swelling ratio during annealing. Specifically, higher SR values correlated with increased neck defect density, while lower SR values led to a greater disclination density. This observation can be attributed to the differing thermodynamic properties of these defects. The swelling of the BCP film enhanced thermal fluctuations, which helped eliminate defects. On the other hand, the swelling weakened the effective strength of phase separation, *χ*_eff_*N,* which simultaneously lowered the defect formation energy, *E*_d_, and the energy barrier, *E*_b_, for defect annihilation [50,51,52]. Theoretically, defect density can be predicted by $n_{d}\approx {a_{c}}^{-2}\exp(-E_{d}/kT)$; *a*_c_ is the size of the defect core, so a high SR benefits the rising of the defect density *n*_d_ due to decreased *E*_d_ [52]. High SR favors the defect annihilation process due to the increased diffusivity and decreased *E*_b_. Figure 3 illustrates the energy landscapes for different defect types as the SR decreased. For neck defects (defect A), at a high SR, both *E*_b_ and *E*_d_ were small, even comparable to thermal fluctuations, resulting in a high defect density due to the minimal energy difference between the initial and final states. As the SR decreased, the density of these defects diminished due to weakened fluctuations and an increased *E*_b_. Disclinations (defect B) exhibited a significantly higher *E*_b_ than neck defects. At a high SR, they can be annihilated through chain reorganization. However, as the SR decreased, their density rose due to the increased *E*_b_ and reduced chain mobility. In the case of 30 nm PS_62k_-*b*-P2VP_26k_ thin films annealed in THF, a high SR of 2.23, while beneficial for disclination annihilation and long-range order enhancement, resulted in a proliferation of neck defects that disrupted this order (Figure 2a). At a lower SR of 1.99, the increased *E*_b_ hindered disclination annihilation, leading to higher defect densities (Figure 2c). Consequently, achieving long-range-ordered BCP nanopatterns necessitates selecting optimal SR conditions that balance sufficient chain mobility with defect formation and annihilation dynamics [53].

**Figure 4:**
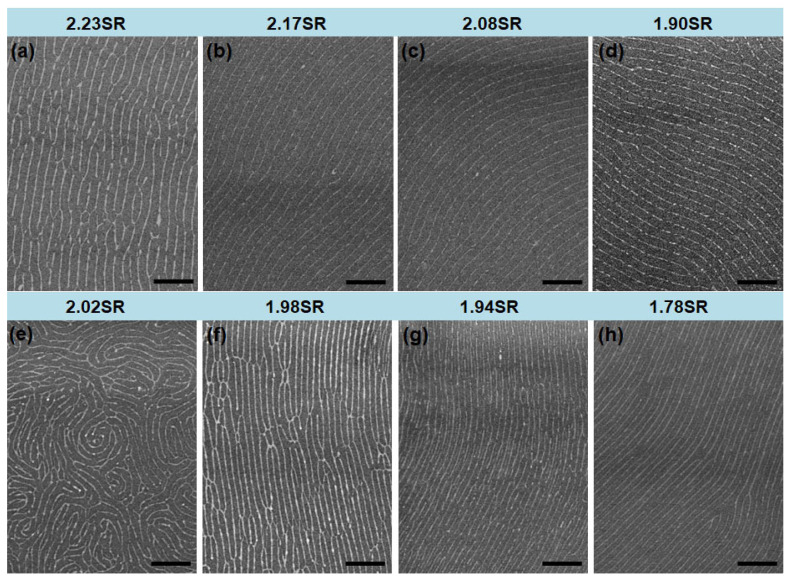
SEM images of the Pt nanopatterns generated from (**a**–**d**) PS_62k_-*b*-P2VP_26k_ and (**e**–**h**) PS_30k_-*b*-P2VP_12.5k_ thin films annealed in THF vapor for 180 s at 22 °C under different equilibrium SRs. Scale bar: 200 nm.

Figure 4 presents representative scanning electron microscope (SEM) images of platinum (Pt) nanopatterns derived from PS-*b*-P2VP thin films annealed in THF for 180 s at varying equilibrium SRs. These Pt nanostructures exhibited a precise replication of the block copolymer (BCP) morphology, thereby enabling effective characterization through SEM. At an equilibrium SR of 2.23 (Figure 4a), interconnected Pt nanowires were observed, mirroring the neck defects present in the PS_62k_-*b*-P2VP_26k_ film (Figure 2a). Long-range-ordered Pt nanowires were achieved at equilibrium SR values of 2.17 and 2.08 (Figure 4b,c). Conversely, reducing the equilibrium SR to 1.90 resulted in Pt nanowires exhibiting a marked increase in disclination density (Figure 4d). For the PS_30k_-*b*-P2VP_12.5k_ BCP, the defect type and density displayed a similar trend with the SR variation (Figure 4e–h). Based on these observations, the optimal SR regimes were determined to be approximately 2.17 to 2.08 for PS_62k_-*b*-P2VP_26k_ and 1.94 to 1.78 for PS_30k_-*b*-P2VP_12.5k_ when annealed in THF. These findings highlighted the essential importance of attaining an optimal SR in the fabrication of highly ordered block copolymer (BCP) thin film morphologies. The optimal solvent annealing conditions were system-specific, dictated by the unique thermodynamic properties of each BCP system. To further validate the efficiency of the annealing strategy, identical optimal annealing conditions were applied to cylinder-forming PS_67k_-*b*-PtBuA_32k_ and PS_67k_-*b*-PtBuA_32k_/PtBuA blends, using THF at optimized SR values. Notably, these samples exhibited well-ordered morphologies after a 180 s annealing. The accelerated development of ordered structures demonstrated the reliability and efficiency of the annealing protocol in promoting the self-assembly of block copolymers and their blends (Supporting Information, S3).

**Figure 5:**
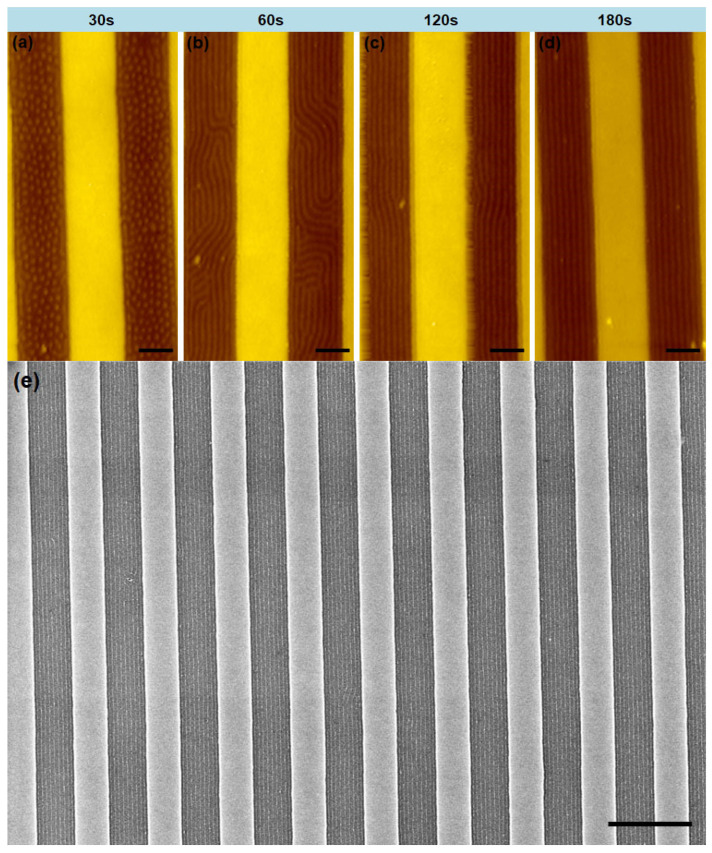
(**a**–**d**) Typical AFM height images of PS_62k_-*b*-P2VP_26k_ nanopatterns as a function of time after annealing in THF vapor with an equilibrium SR of 2.08: (**a**) 30 s, (**b**) 60 s (**c**) 120 s, and (**d**) 180 s. (**e**) SEM images of Pt nanowires derived from the PS_62k_-*b*-P2VP_26k_ nanopattern template after 180 s of annealing. (**a**–**d**) Scale bar: 200 nm (**a**–**d**), (**e**) scale bar: 1 μm.

In addition to solvent annealing on flat substrates, we also explored epitaxial directed assembly of BCP films within 416.5 nm wide trenches on topologically patterned substrates. Figure 5a–d illustrates the PS_62k_-*b*-P2VP_26k_ film morphology after annealing in THF at 22 °C with an SR of 2.08 for 30, 60, 120, and 180 s, respectively. After 30 s of annealing, a mixed morphology of vertically and parallel-oriented cylinders was observed. Notably, only a few parallel cylinders appeared along the groove edges, indicating that the ordering process was preferentially initiated from these edge regions (Figure 5a). Following 60 s of annealing, parallel cylinders formed but exhibited numerous structural defects (Figure 5b). With further prolonged annealing, these defects were largely eliminated, and parallel cylinders aligned exclusively along the trench walls, demonstrating long-range order (Figure 5c,d). Figure 5e presents a large-area scanning electron microscope (SEM) image of highly ordered platinum (Pt) nanowires, derived from the PS_62k_-*b*-P2VP_26k_ nanopattern after 180 s of annealing, which demonstrated the successful fabrication of parallel Pt nanowires extending over several micrometers on the patterned substrate. These results suggest that long-range-ordered nanostructures can be achieved within minutes, provided that the appropriate SR and solvent vapor pressure are precisely controlled (Supporting Information, S4).

## 4. Conclusions

In summary, we achieved the rapid assembly of block copolymer (BCP) thin films by enhancing the swelling kinetics during solvent annealing. This optimized annealing conditions and accelerated solvent vapor equilibration, enabling precise vapor pressure regulation without modifying solvent volume or annealing temperature. Additionally, precise control of the BCP film’s swelling ratio was achieved, revealing that both defect morphology and density are strongly correlated with the swelling ratio. Moreover, a systematic relationship between defect density and defect type was observed. This solvent annealing-based strategy for rapid BCP film assembly not only serves as a reliable alternative to conventional methods that rely on solvent volume or temperature modulation but also demonstrates considerable potential for scalable, high-throughput, and cost-effective manufacturing.

## Figures and Tables

**Table 1:** The different swelling ratios (SRs) of PS_62K_-*b*-P2VP_26K_ and PS_30K_-*b*-P2VP_12.5K_ films, respectively, determined by the different spacer heights when annealed with THF.

	Spacer Height (mm)	Swelling Ratio ^1^		Spacer Height (mm)	Swelling Ratio ^1^
PS_62K_-*b*-P2VP_26K_	0.05	2.23	PS_30K_-*b*-P2VP_12.5K_	0.05	2.02
	0.10	2.21		0.10	1.98
	0.15	2.17		0.15	1.94
	0.20	2.08		0.20	1.90
	0.25	1.99		0.25	1.87
	0.30	1.97		0.30	–
	0.40	1.90		0.40	1.78
	0.50	1.77		0.50	–

^1^ Swollen film thickness *H* divided by initial film thickness *H*_0_, *H*/*H*_0_.

## Data Availability

The original contributions presented in the study are included in the article/supplementary material, further inquiries can be directed to the corresponding authors. [insert reason here].

## Supplementary Materials

The following supporting information can be downloaded at: https://www.mdpi.com/article/10.3390/polym17091242/s1, S1: Theoretical prediction of the viscosity and glass transition temperature of the polymer in the molten state and concentrated solution; S2: The SR-dependent morphologies as a function of time for PS-*b*-P2VP BCPs; S3: Morphologies of PS_67k_-*b*-PtBuA_32k_/PtBuA films under optimized annealing condition; S4: Nanopatterns of PS-*b*-P2VP BCP films annealed on topological patterned substrates. Reference [54] is cited in the supplementary materials.
