# Supplementary material for: Rapid Assembly of Block Copolymer Thin Films via Accelerating the Swelling Process During Solvent Annealing
Source: Polymers (Basel). 2025 May 2;17(9):1242. doi: 10.3390/polym17091242 (PMC12074153; doi:10.3390/polym17091242)
Supplement: Supplementary file 1 [file polymers-17-01242-s001.zip › polymers-3573194-supplementary.pdf]

Supporting Information

Table of Contents

S1 Theoretical prediction of the viscosity and glass transition temperature of the polymer in molten state and concentrated solution

S2 SR-dependent morphologies as a function of time for PS-*b*-P2VP BCPs

S3 Morphologies of PS_{67k}-*b*-$PtBuA_{32k}$/PtBuA films under optimized annealing condition

S4 Nanopatterns of PS-*b*-P2VP BCP films annealed on topological patterned substrate

S1. Theoretical prediction of the viscosity and glass transition temperature of the polymer in molten state and concentrated solution

It is difficult to estimate the viscosity of block copolymer under ontic state and solution state, so we use homopolymer (polystyrene, PS) as a model to compare the viscosity under the two different states, as well as to check the effectiveness of thermal annealing and solvent annealing in reducing the viscosity.

A. Polymer in molten state

Viscosity and molecular weight. Herein the molecular weight of PS is 100kDa (PS_{100k}), larger than the critical entanglement molecular weight 35kDa, the relationship between molecular weight and viscosity follows: $\eta \propto M^{3.4}$, hence the system viscosity η_0 can be calculated from η_{cr}:

$$\log \eta_0 = \log \eta_{cr} + 3.4 \log \frac{M_W}{M_{cr}}$$

Viscosity and temperature. When the temperature is higher than the viscous flow temperature, $\eta = Be\frac{\Delta E_\eta}{RT}$, ΔE_η is the flow activation energy, B is a constant related to the system. Based on this, Van krevelen and Hoftyzer deduced that system viscosity could be estimated by the following calculation equation[54]:

$$\log \eta_{cr}(T) = \log \eta_{cr}(\infty) + \frac{E_\eta(\infty)}{2.3RT}$$

$$\log \eta_{cr}(\infty) = \log \eta_{cr}(1.2T_g) - A$$

Incorporating the aforementioned relationship, it is clearly shown that the system viscosity can be predicted by:

$$\log \eta_0(T) = \log \eta_{cr}(1.2T_g) - A + \frac{E_\eta(\infty)}{2.3RT} + 3.4 \log \frac{M_W}{M_{cr}}$$

For PS, A=8.2, $E_\eta(\infty)$=59kJ/mol, $\log \eta_{cr}(1.2T_g)$=1.8Pa·s. The calculated theoretical results are shown in Figure S1a.

B. Polymer in concentrated solution

According to Bueche theory, the glass transition of the plastified polymer can be predicted by the following equation:

$$T_g = \frac{\phi_P T_{gp} + \phi_S K T_{gs}}{\phi_P + K\phi_S}$$

In the equation, Φ_P and Φ_S refers to the volume fraction of polymer and solvent respectively. T_{gp} and T_{gs} refers to the glass transition temperature of pure polymer and solvent respectively. K is a constant, $K \approx \frac{\alpha_{1S} - \alpha_{gS}}{\alpha_{1P} - \alpha_{gP}}$, α_1 is the expansion coefficient above the T_g, α_g is the expansion coefficient below the T_g. K is always taken as 2.5 when it can not be accurately calculated. For the toluene solution of PS_{100k}, the glass transition of toluene is 106K, and the glass transition temperature for pure PS adopts 373K.

For the concentrated polymer solution, the relationship between the system viscosity following $\eta \propto C^{\alpha} M^{\beta}$, β is closed to 3.4, while α always ranges from 4.0 to 5.6, with a average value about 5.1. To facilitate the calculation, herein α is taken as 5.

$$\log \eta_{cr} = \log \eta_{cr}^{*} + 5 \log \phi_p$$

$$\log \eta_0 = \log \eta_{cr} + 3.4 \log \frac{M_W}{M_{cr}}$$

In this equation, η_{cr}^* is the system viscosity with a concentration of 1 at the critical entanglement molecular weight.

The system viscosity can be deduced from the relationship between the viscosity and temperature:

$$\log \frac{\eta_{cr}(T)}{\eta_{cr}(1.2T_g)} = A(\frac{T_g}{T} - 1)$$

The value of T_g in the equation should adopt the glass transition temperature of the concentrated solution, and T is the real temperature (room temperature, 25°C). So the system viscosity can be calculated from the following equation:

$$\log \eta(M,T,C) = \log \eta_{cr}(1.2T_g) + A(\frac{T_g}{T} - 1) + 5 \log \phi_p + +3.4 \log \frac{M_W}{M_{cr}}$$

For PS, A=8.2, $E_{\eta}(\infty)$=59kJ/mol, $\log \eta_{cr}(1.2T_g)$=1.8Pa·s. The results are shown in Figure S1b.

Clearly, the polymer molecular diffusivity can be dramatically enhanced by the introduction of solvent molecules simultaneously as diluter and plasticizer. For example, the ontic viscosity of PS with molecular weight of 100 kDa is about 47 Pa.s

at 200 °C, while the viscosity of PS toluene solution (50 wt%) at room temperature 20 °C is about 0.046 Pa.s and a depressed T_g about -89 °C.

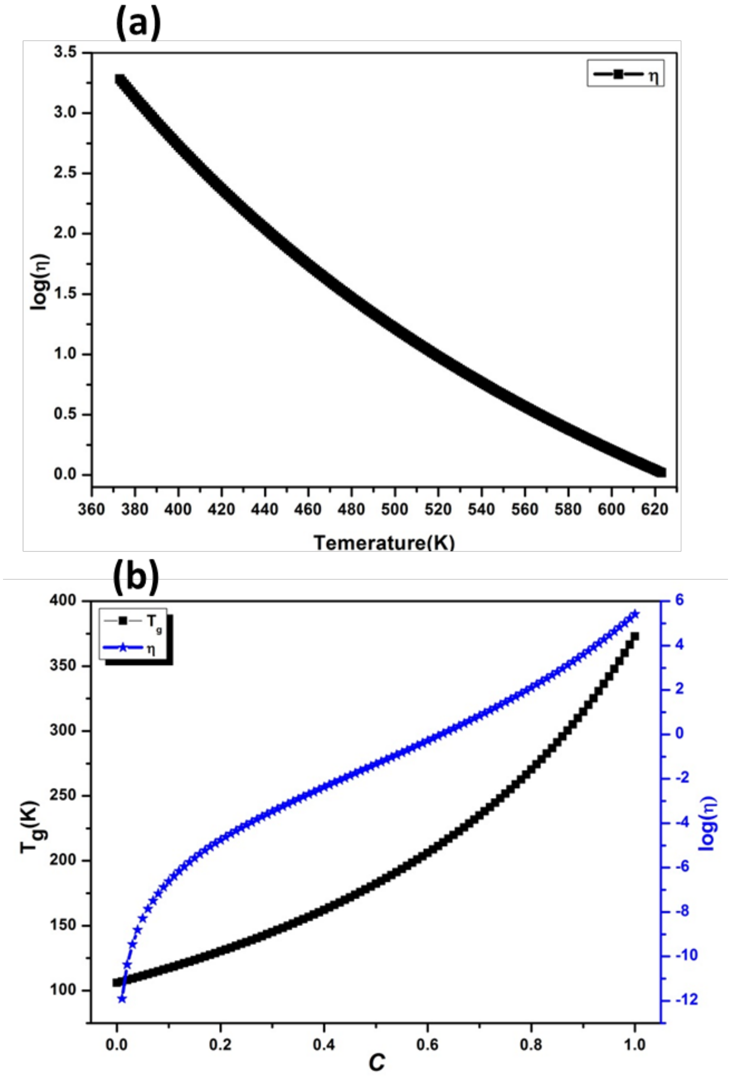

Figure S1. (a) Plot of the viscosity of Polystyrene (PS) for various temperature. (b) Plots of the glass transition temperature (T_g) and viscosity of PS solution in toluene for various concentration at 298K. The PS molecular weight was both set as 100 kDa.

References

(54) Krevelen, D.W.V. *Properties of Polymers. Their Correlation with Chemical Structure; Their Numerical Estimation and Prediction from Additive Group Contributions*, 4th ed.; Elsevier: Amsterdam, The Netherlands, 2009

S2. The SR-dependent morphologies as a function of time for PS-*b*-P2VP BCPs

Figure S2 presents the surface morphology of PS-b-P2VP thin films annealed in tetrahydrofuran (THF) vapor at varying equilibrium swelling ratios for 30, 60, and 120 seconds, respectively. The atomic force microscopy (AFM) measurements were performed over an extended scanning area compared to those presented in the main text. The characteristic domain spacing was quantitatively measured from the AFM images.

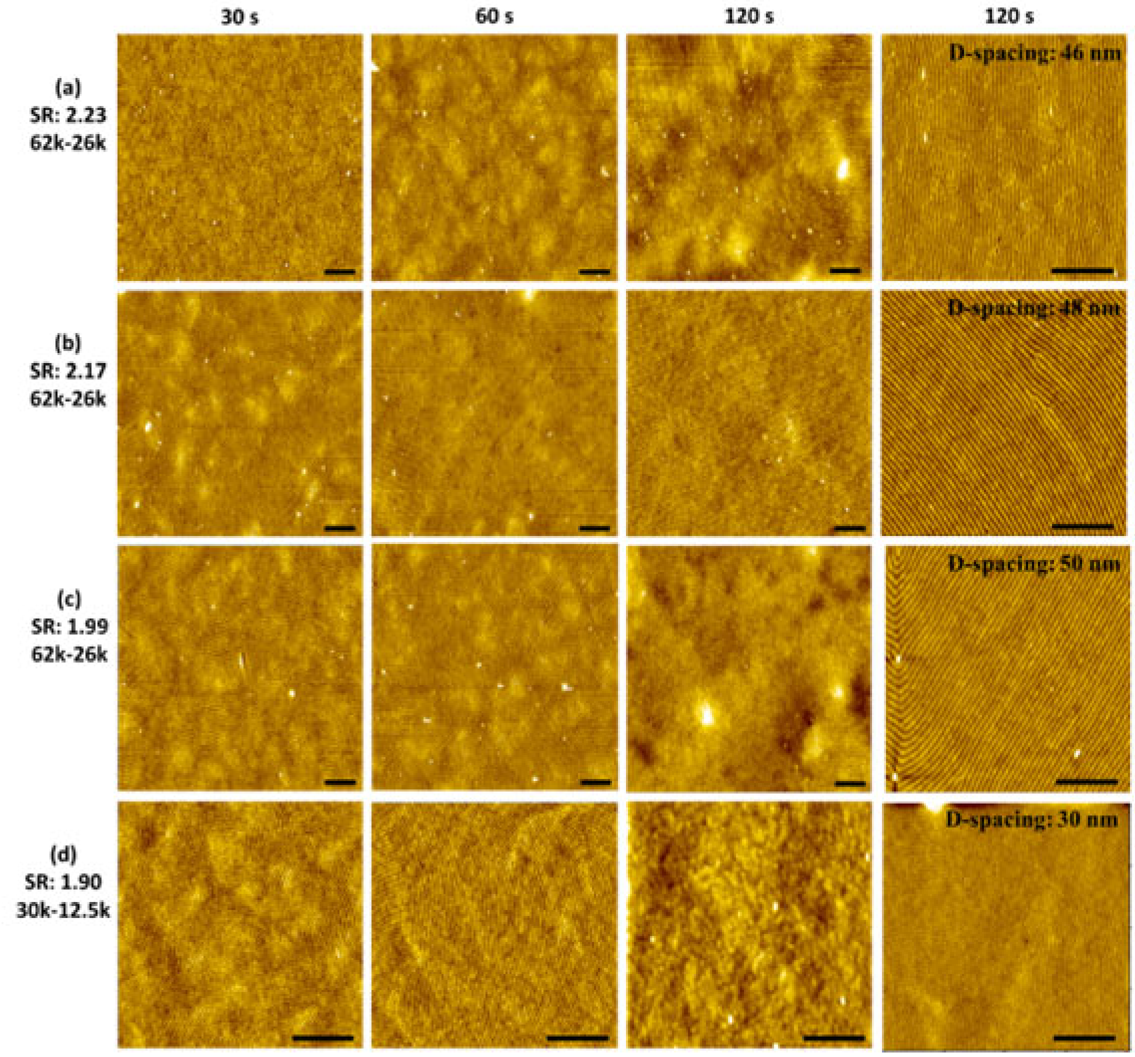

Figure S2. Typical atomic force microscope (AFM) height images of the PS-*b*-P2VP thin films annealed by THF with different SR for 30s, 60s and 120 s, respectively at 22 °C: (a-c) 30 nm PS_{62k}-*b*-$P2VP_{26k}$ films annealed with different equilibrium SR. (d) 20 nm PS_{30k}-*b*-$P2VP_{12.5k}$ films annealed with equilibrium SR of 1.90. Scale bar: 500 nm..

S3. Morphologies of PS_{67k}-*b*-$PtBuA_{32k}$/PtBuA films under optimized annealing condition

The annealing experiment were also performed on blends of PS_{67k}-*b*-$PtBuA_{32k}$ and PtBuA homopolymer by varying the PtBuA composition. Figure S3 shows the typical AFM height images of 25nm PS_{67k}-*b*-$PtBuA_{32k}$ thin films with different composition of PtBuA homopolymer after annealing in THF for 180s with equilibrium SR of 1.90 at 22 °C. Ordered perpendicular cylinders were observed for pure PS_{67k}-*b*-$PtBuA_{32k}$ BCP thin film (Figure S3b), while ordered parallel cylinder were observed for PS_{67k}-*b*-$PtBuA_{32k}$ BCP blended with 5% (Figure S3c) or 10% PtBuA (Figure S3d) after solvent vapor annealing.

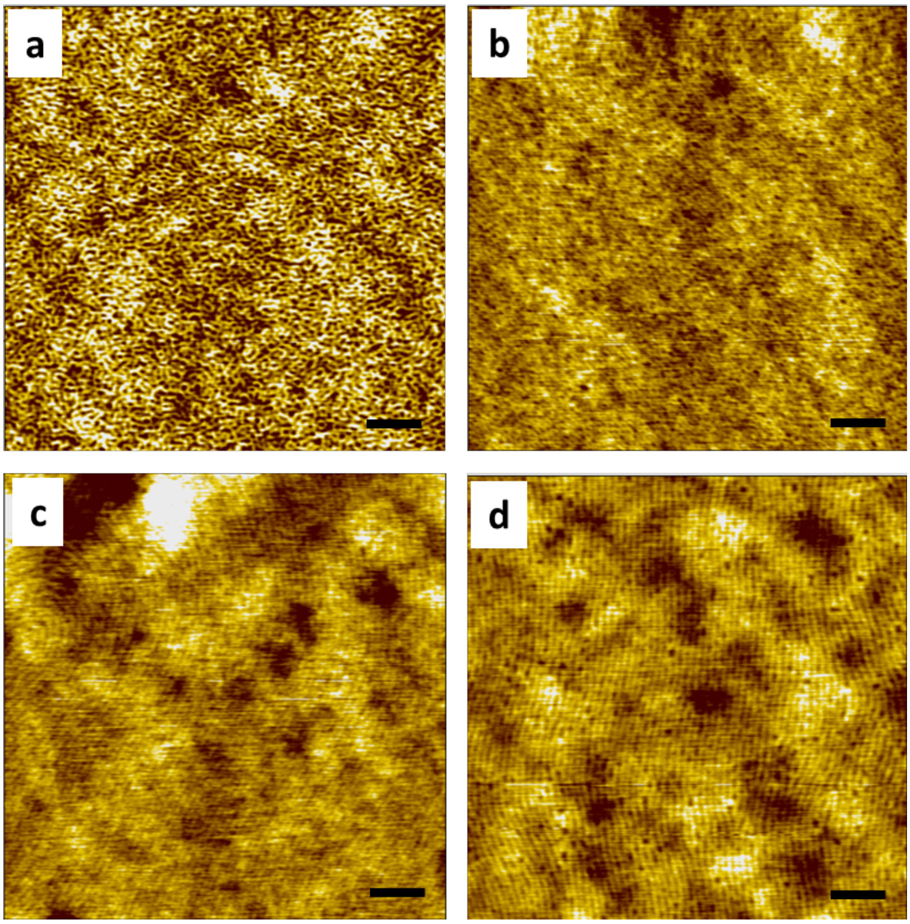

Figure S3. Typical AFM height images of PS_{67k}-*b*-$PtBuA_{32k}$/PtBuA thin film morphology with different PtBuA composition before (a) and after annealing (b-d) in THF for 180 s with equilibrium SR of 1.90 at 22 °C: (a) as-cast PS_{67k}-*b*-$PtBuA_{32k}$/PtBuA thin films before annealing; (b) PS-*b*-PtBuA/0%PtBuA; (c) PS-*b*-PtBuA/5%PtBuA; (d) PS-*b*-PtBuA/10%PtBuA. Scale bar: 400 nm.

S4. Nanopatterns of PS-*b*-P2VP BCP films annealed on topological patterned substrates

Figure S4 illustrates the PS_{62k}-*b*-$P2VP_{26k}$ film morphology as a function of time after annealing in THF with SR of 1.78. After 180 s annealing, the parallel cylinders aligned exclusively along the trench walls, demonstrating a long-range order. Figure S5 displayed the SEM images of Pt nanostructures derived from PS_{62k}-*b*-$P2VP_{26k}$ and PS_{30k}-*b*-$P2VP_{12.5k}$ films, respectively. A variety of ordered cylindrical microstructures were observed on the patterned substrates. As the film thickness was increased, the formation of ordered double-layer cylindrical structures was detected (Figure S5b). Furthermore, the development of vertically oriented cylindrical domains was achieved following toluene vapor annealing (Figure S5d).

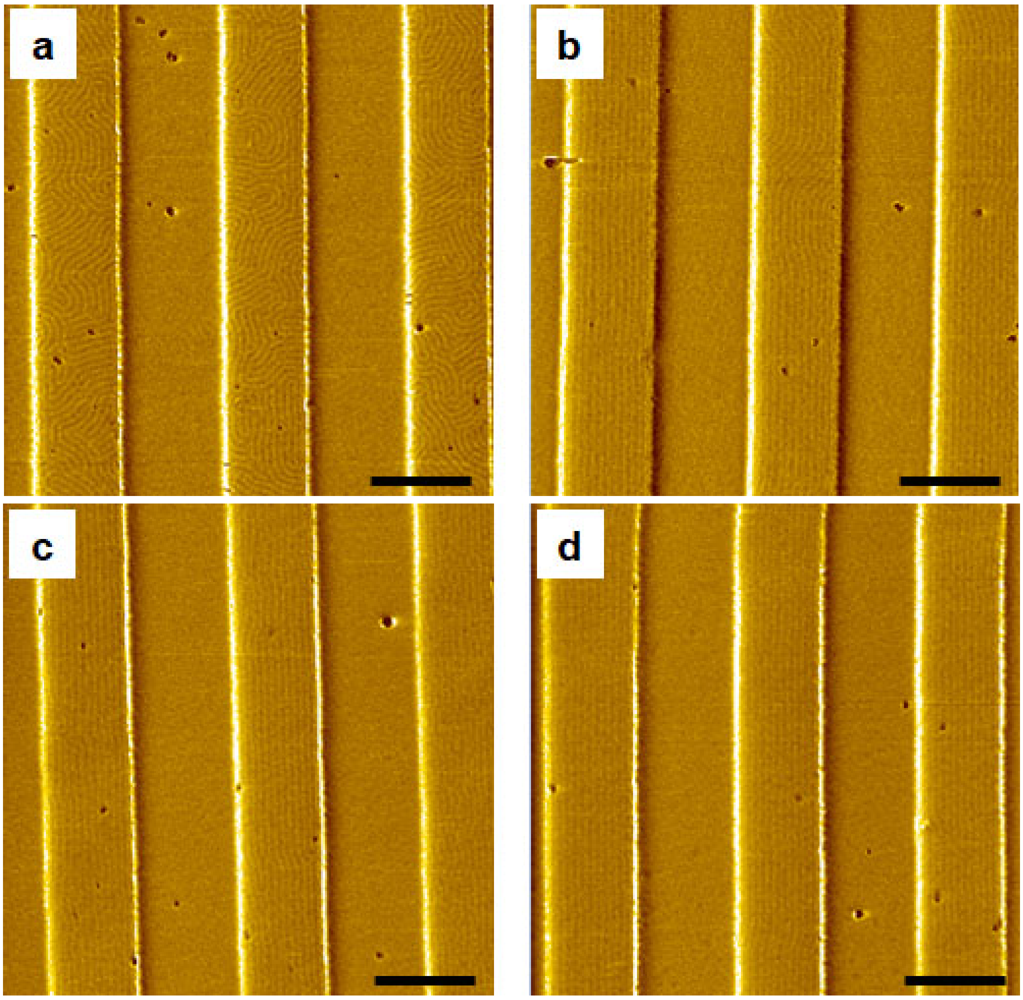

Figure S4. Typical AFM phase images of PS_{30k}-*b*-$P2VP_{12.5k}$ nanopatterns as a function of time after annealing in THF vapor with equilibrium SR of 1.78: (a) 30 s, (b) 60 s (c) 120 s and (d) 180 s. Scale bar: 400 nm.

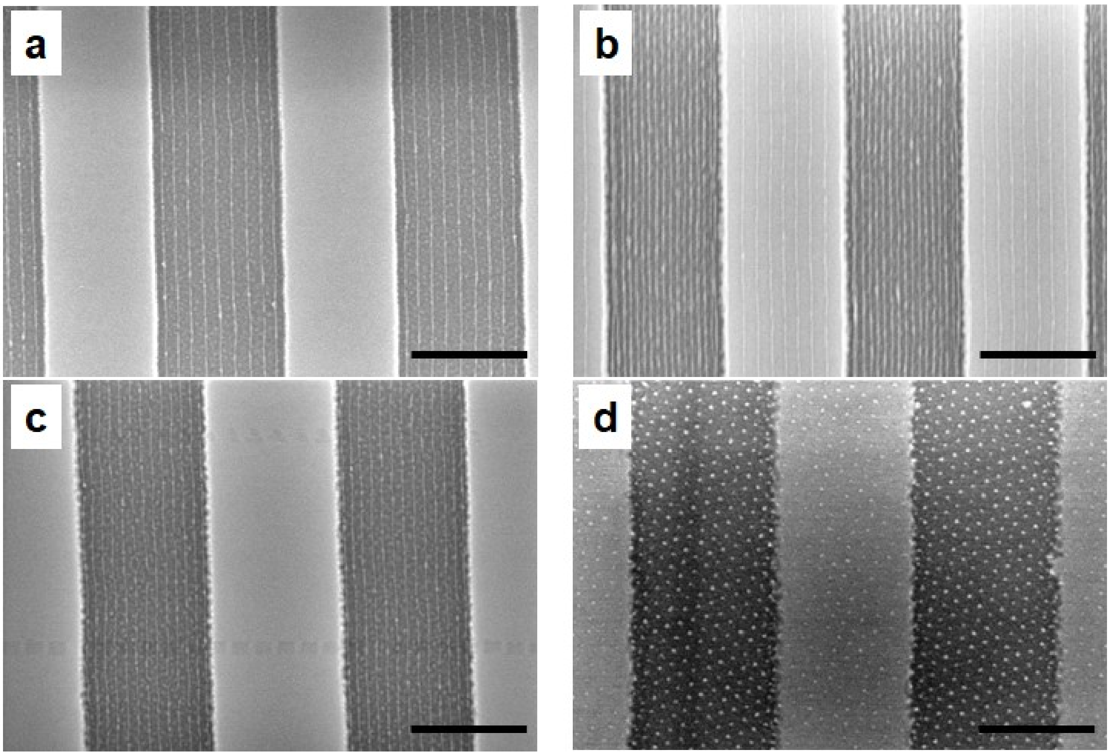

Figure S5. SEM images of the Pt nanostructure fabricated from PS-*b*-P2VP after annealing in (a-c) THF or (d) toluene for 180 s at the optimal SR conditions: (a) monolayer of PS_{62k}-*b*-$P2VP_{26k}$ parallel cylinder, (b) bilayer of PS_{62k}-*b*-$P2VP_{26k}$ parallel cylinder, (c) monolayer of PS_{30k}-*b*-$P2VP_{12.5k}$ parallel cylinder, (d) perpendicular PS_{62k}-*b*-$P2VP_{26k}$ cylinder. Scale bar: 400 nm.
